# What are the benefits of early patient contact? - A comparison of three preclinical patient contact settings

**DOI:** 10.1186/1472-6920-13-80

**Published:** 2013-06-03

**Authors:** Marjorie D Wenrich, Molly B Jackson, Ineke Wolfhagen, Paul G Ramsey, Albert JJ Scherpbier

**Affiliations:** 1 Office of the CEO, UW Medicine and Executive Vice President for Medical Affairs, University of Washington, Box 356350, Seattle, WA 98195-6350, USA; 2 Department of Medicine, University of Washington School of Medicine, Box 356429, Seattle, WA 98195-4328, USA; 3 Faculty of Health, Medicine and Life Sciences, Maastricht University, P.O. Box 616, 6200, MD, Maastricht, the Netherlands

## Abstract

**Background:**

Despite increasing attention to providing preclinical medical students with early patient experiences, little is known about associated outcomes for students. The authors compared three early patient experiences at a large American medical school where all preclinical students complete preceptorships and weekly bedside clinical-skills training and about half complete clinical, community-based summer immersion experiences. The authors asked, what are the relative outcomes and important educational components for students?

**Methods:**

Medical students completed surveys at end of second year 2009–2011. In 2009, students compared/contrasted two of three approaches; responses framed later survey questions. In 2010 and 2011, students rated all three experiences in relevant areas (e.g., developing comfort in clinical setting). Investigators performed qualitative and quantitative analyses.

**Results:**

Students rated bedside training more highly for developing comfort with clinical settings, one-on-one clinical-skills training, feedback, active clinical experience, quality of clinical training, and learning to be part of a team. They rated community clinical immersion and preceptorships more highly for understanding the life/practice of a physician and career/specialty decisions.

**Conclusions:**

Preclinical students received different benefits from the different experiences. Medical schools should define objectives of early clinical experiences and offer options accordingly. A combination of experiences may help students achieve clinical and team comfort, clinical skills, an understanding of physicians’ lives/practices, and broad exposure for career decisions.

## Background

The medical education community has increasingly emphasized the value of early patient contact experiences for preclinical medical students. In the influential volume *Educating Physicians* from the Carnegie Foundation, the authors called for early clinical immersion to help integrate skills and knowledge in preparation for practice [1].

Dornan defined early clinical experience as pre-clerkship experiences with authentic patient contact in a clinical context that enhances learning [2]. These experiences frequently take the form of community-based preceptorships. Objectives may include: developing comfort with patients; basic clinical-skills training; promoting career interest in primary care and specialty understanding; encouraging active learning in preclinical settings; and reducing the “shock of practice” that some students experience as they enter clerkships [3,4]. Data suggest that early clinical exposure can make basic science curricula more relevant [5] and help prepare students for clerkships [6].

Dornan’s review suggests that early experiences help students socialize to medicine, strengthen learning and skills acquisition, and make learning more relevant [2]. Yardley’s follow-up study suggests that early experiences help students understand and align with patient and community perspectives [7].

Theory supports use of early patient experiences [8,9]. Concrete experience is essential to learning; active experience, as opposed to observation, provides the greatest impact on learning and seeing patients independently may be the optimal setting for achieving positive learning outcomes [9]. Important in early patient encounters, Ottenheijm and colleagues argue, is continuous supervision, reflection between student and supervisor, and timely feedback. Early clinical experiences may provide legitimate peripheral participation, gradually drawing students into the workplace [10].

Early clinical experiences may be associated with better academic performance [11], career interest in relevant specialties [5], improvement in the “shock of practice” as students transition into clinical settings [3], and greater comfort entering clerkships [12]. While a recent study showed no differences between pre-clerkship teaching formats for outcomes in clerkships, none of the teaching formats involved direct contact or involvement with real patients [13].

Yardley noted that two specific questions have not yet been fully answered: ‘How and why do particular early experience interventions lead to specific learning outcomes?’ and ‘What is essential to make early authentic experience a more effective process?’ [7] Hopavian and colleagues have noted the lack of national guidance in the United Kingdom that supports a minimum quantity of patient contact or specific educational purpose in the early years of U.K. basic medical training [14]. As medical schools move toward competency-based approaches [15] and other curricular models that attempt to identify and shape student experience and to define what students obtain from each stage of learning [16], it is important to understand what competencies students receive from preclinical patient experiences. This can help assess the relationship between curricular objectives and outcomes and will help guide choices for early clinical programs.

We used a mixed-method, iterative approach to compare three types of preclinical patient contact at a medical school where many students complete all three, and all complete at least two of the three. In assessing and comparing students’ experiences within these approaches, we asked, what are the relative benefits for students from different types of early patient contact and what are the important educational components for students from each?

## Methods

First-year medical students at the University of Washington School of Medicine must complete at least one community preceptorship and are encouraged to complete one or more in second year as well. These preceptorships consist of half-day, unstructured experiences in the offices of community physicians; students can select the specialty of the preceptor with whom they work. The preceptorships are arranged and monitored by individual departments. Students’ involvement varies considerably, from shadowing the preceptor to active participation (i.e., taking history, performing physical exam under supervision).

Approximately half of medical students complete an optional four-week full-time community immersion in a rural or urban underserved setting called the Rural/Underserved Opportunities Program (R/UOP) between their first and second years [17]. In R/UOP, students live in the community of their preceptor (typically a rural community) and get to know the community in addition to the preceptor’s practice; the immersion experience lacks formal structure in terms of clinical objectives and expected clinical outcomes. As with preceptorships, the level of student involvement varies considerably, depending on the preferences of the preceptor. Students may shadow the R/UOP preceptor (often including other preceptors in the community) or may be more actively involved in simple tasks such as history-taking and learning physical exam under supervision. Preceptors receive extensive orientation materials, including how to introduce students into one’s practice, administrative issues such as liability, information about students’ coursework to date, etc. A standard evaluation form is used to assess student performance on characteristics such as student’s practice management skills, clinical skills and attitude.

Finally, throughout second year, medical students spend one-half day weekly with a dedicated faculty mentor and a small group of peers learning clinical skills at the bedside through the Colleges program, a structured curricular program with an established group of faculty mentors [18]. Each week, two of the six students in each small group are assigned to independently complete a full history and physical on a hospitalized patient. Patients seen by the students are not from the College mentors’ practices; rather, they are patients who are selected on the morning of the tutorial at local medical centers by patient interview coordinators who are trained to identify and approach patients about voluntary participation in this educational activity.

The students’ College mentor moves back and forth between the two students as they work with patients, observing, taking notes and offering suggestions. The six students then convene with the mentor for student oral case presentations at the bedside. Students who are observing that morning then ask follow-up questions of the patient about their past medical history and presenting concern; the College mentor guides the bedside discussion and often demonstrates interview and physical exam techniques. Patients are invited to participate actively in these bedside discussions. After the session, each interviewing student completes a full write-up of their patient case and submits it to the College mentor for review. Over the course of a year, each student performs a full history and physical, oral case presentation, and write-up on six patients (and observes an additional 30 bedside presentations by their peers). College mentors give explicit constructive feedback during College mornings, as well as in several one-on-one meetings with students throughout the year.

The stated goals of first- and second-year preceptorships are to: provide early patient contact that allows students to become more comfortable in patient encounters; provide opportunities to observe and practice physician-patient communication; and provide student awareness about the practice of a particular specialty. The stated goals of R/UOP are to: provide students with early exposure to the challenges and rewards of primary-care medicine in a rural or urban underserved setting; promote positive attitudes toward rural and urban underserved community medicine; and learn how community healthcare systems function. The stated goals of the Colleges are to provide systematic clinical-skills training and early patient contact in a consistent pre-clinical experience.

In the past, required first-year and optional first- and second-year community preceptorships were the primary venue for early patient exposure and pre-clerkship clinical-skills training (apprenticeship model), augmented by classroom and standardized-patient training; there was minimal control over preceptorship content in multiple settings. The Colleges program was developed to build basic clinical skills in the context of real patients, provide exposure to patients, and help students develop a long-term mentoring relationship with a faculty member (experience-based learning).

Having three different early patient experiences in the same medical school permits assessment of their respective roles for students. We assessed domains common to the three settings and asked whether the three types of early patient contact achieve similar or unique outcomes across those domains from students’ perspectives. If there are common experiences in the different settings, they may be redundant or duplicative; however, if the experiences have unique aspects of complementary value to students, encouraging multiple early patient care experiences may be useful for other medical schools insofar as the experiences align with curricular goals. In order to focus on competencies, we excluded areas such as motivation, confidence building, and other affective domains that have been assessed elsewhere and that are less likely to fall within curricular objectives [16].

### Instruments

Data were collected across three consecutive years from students during spring of their second year. Areas examined were common to all three settings and relevant to students’ future needs. The process of item development was iterative; results from each year informed questions posed in the subsequent year. The data sets were:

1. 2009 survey: We asked students to compare and contrast their preceptorship experiences with training experience in the Colleges program. Students were specifically asked: Please comment on similarities and differences between clinical-skills training during preceptorships and through the Colleges in second year. The response option was open-ended, with no prompts. Students were asked to identify preceptorships they completed and whether they completed R/UOP.

2. 2010 and 2011 surveys: Based on the domains identified in the 2009 survey and programmatic objectives for early patient experiences, we asked students in 2010 to rate their early patient experiences (1^st^ year preceptorships, 2^nd^ year preceptorships, R/UOP, and Colleges) on the following using a Likert scale (1=not at all useful to 5=extremely useful): developing comfort in clinical settings, receiving one-on-one clinical-skills training, receiving feedback on clinical performance, and understanding the daily life of a physician. Students were also asked to rate how passive or active their clinical experience was in each setting and the overall quality of their clinical training in these settings. Students were asked to identify preceptorships completed and whether they completed R/UOP. Because the Colleges program was relatively new at the time and being closely evaluated, we asked what single aspect of the Colleges is most important to them, using an open-ended format, to ascertain if other aspects of the College experience not asked about were important for students. In 2011, we repeated a portion of the 2010 survey but added two additional questions to address areas missing from the 2009 survey and of likely importance to the student experience based on the literature and student comparisons of preceptorships and the Colleges. Due to survey space limitations, we deleted questions asked in 2010 to allow the two new questions in 2011. The new dimensions were: learning to be part of a team and making career/specialty decisions. The deleted dimensions were receiving feedback, passive/active experience and overall quality of clinical training (the latter was seen as comparable to one-on-one clinical skills training). Because no statistically significant differences were found in ratings of first- and second-year preceptorships in 2010, we combined them into a single category of preceptorships in 2011. Students were asked to list preceptorships completed. In 2011, the same Likert scale was used as in 2010.

### Participants and data collection

Second-year medical students completed 2009 and 2010 surveys in debriefing sessions at the completion of a required objective structured clinical examination (OSCE) held each May. In 2011, students completed the survey in June as part of an end-of-year program evaluation survey. Participation was voluntary.

### Analyses

Open-ended comments about similarities and differences between preceptorships and Colleges were analyzed using open coding. Comments were reviewed and grouped into themes, first by one investigator (MDW) and then independently by a second investigator (MBJ) to validate themes in initial coding. Codes were entered into a qualitative data entry program, Atlas Ti, and reviewed for agreement and to resolve disagreements between the two investigators’ coding. Disagreements were discussed and consensus developed. For the open-ended question asked in 2010, “What single aspect of the Colleges is most important to you,” a single investigator (MDW) reviewed and assigned codes to responses. For the 2010 and 2011 survey results, data were entered into SPSS and descriptive statistics were prepared. Analysis of variance was performed to compare responses, with type of experience (preceptorship, R/UOP, and College group) as independent variables. Post-hoc comparisons were completed using Fisher’s Least Significant Difference Test. The alpha was set at .05.

### Ethical approval

This study received ethical review by the University of Washington Human Subjects Division and received approval as an exempt study.

## Results

Response rates for the three surveys were:

2009: 191 surveys completed out of 191 OSCE examinees: 100%

2010: 218 surveys completed out of 218 OSCE examinees: 100%

2011: 145 surveys completed out of 200 medical students: 73%

### Contrasts between preceptorships and College experience

Table 1 shows the most frequent contrasts/comparison in themes identified from open-ended comments in 2009 by students concerning clinical training in community preceptorships compared with the Colleges.

**Table 1:** 2009 comparisons of the College experience with community preceptorships identified by second-year medical students

**Comment category**	**Dominant comparison**	**Sample quotes**
Passive versus active learning	Colleges active, hands-on	“Preceptorships haven’t been much more than shadowing…College actually provides the opportunity to DO what I’m learning.”
	Preceptorships shadowing	
Comprehensive versus focused	Colleges comprehensive	“I learned a lot more specialized information in my preceptorship versus general information in [Colleges]. Preceptorships offer a better intro into the clinical world and short focused histories and physicals. Both were beneficial.”
	Preceptorships specialized, focused	
Structured versus unstructured	Colleges structured, rigorous, formal	“College is very ‘ideal’—follows guidelines/benchmarks…Preceptorships very quick and dirty version of what we learn in the Colleges.”
	Preceptorships unstructured, spontaneous, relaxed	“Colleges are more formalized to cover all bases. In preceptorships, we get lots of exposure to bread & butter, but less variety. I think they are complementary.”
Specific skills areas	Colleges in physical exam and clinical reasoning	“Preceptorship was good for learning the mechanics and how you treat. Colleges good for interviewing, PE, OCP, and clinical reasoning.”
	Preceptorships in techniques, procedures, treatment	
Real life versus academic	Colleges theoretical, academic	“Much more opportunity to practice and receive feedback through Colleges. Much more opportunity to learn specifics of clinical care, realities of practice and exposure to potential career options through preceptorships.”
	Preceptorships real life	

In response to the open-ended question asked in 2010, “What single aspect of the Colleges is most important to you,” the three most frequent responses focused on: 1) patient contact (45 comments); 2) mentor relationship with a faculty member (33 comments); and 3) working in a team/small group setting (33 comments), including development of camaraderie. The second tier of comments focused on skill development: specific skill areas (29 comments), one-on-one teaching from a mentor (24 comments), bedside learning (19 comments) and receiving feedback (19 comments).

### Assessments of learning experiences in specific domains

**Figure 1:**
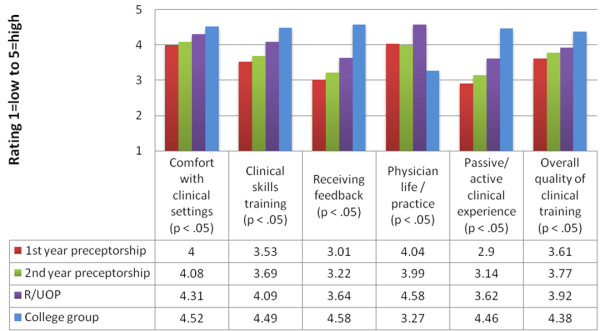
2010 comparison of second-year student ratings of different early patient contact experiences (n=218).

**Figure 2:**
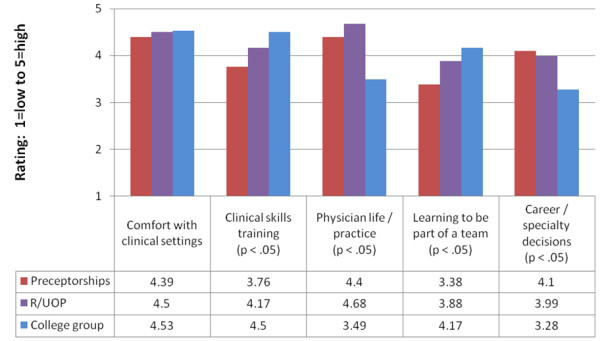
2011 comparison of second-year student ratings of different early patient contact experiences (n=145).

Results from 2010 and 2011 surveys are shown in Figures 1 and 2. In analysis of variance, statistically significant differences were found for all six domains in 2010 and four of five domains in 2011.

For analyses related to *clinical skills training*, students rated the Colleges experience significantly higher than R/UOP and preceptorships for one-on-one clinical-skills development in 2010 and in 2011. They also rated R/UOP significantly higher than preceptorships in both years. For receiving feedback and overall quality of clinical training (asked in 2010), the same pattern was seen: students rated the Colleges significantly higher than both R/UOP and preceptorships and rated R/UOP significantly higher than first-year preceptorships.

For analyses related to *developing comfort with clinical settings*, students rated the Colleges significantly higher than R/UOP and preceptorships in 2010 and also rated R/UOP significantly higher than preceptorships. However, in contrast to 2010, no significant differences were found for the category of developing comfort with clinical settings in 2011. For learning to be part of a team, asked in 2011, students rated the Colleges and R/UOP significantly higher than preceptorships. For having an active compared with passive learning experience asked in 2010, students rated the Colleges significantly higher than both R/UOP and preceptorships and rated R/UOP significantly higher than preceptorships.

For analyses related to *considering and examining career or physician practice characteristics*, a different pattern was seen. For understanding the life of the physician/what practice is like, students rated R/UOP and preceptorships significantly higher than the Colleges in 2010 and 2011, and rated R/UOP significantly higher than preceptorships in both years. For career/specialty decisions asked in 2011, students rated R/UOP and preceptorships significantly higher than the Colleges.

## Discussion

As medical education moves to new curricular models, including competency-based education with defined objectives, benchmarks and outcomes and more standardized approaches, it is important to understand and set objectives for each phase of education [19,20]. While earlier work has defined existing types of early patient experience during the preclinical phase [2], there has been limited attention to the relative values of these experiences for medical students or to the objectives, competencies and outcomes associated with early patient experiences. Understanding the benefits of each will help shape the best experiences in accordance with a curriculum’s objectives.

In a medical school with three different types of early patient contact, we compared the experiences for broad outcome areas: developing comfort in clinical settings, clinical-skills training and receiving feedback, getting to know specialties and considering potential careers, and team acculturation and active engagement. We found that students received different benefits from the different types of experiences. No single experience offered all these potential benefits.

The Colleges small-group experience, under the supervision of a faculty mentor and within a small group of peers, provided benefits compared with community-based preceptorships in clinical-skills training, active learning and team acculturation. Students rated the Colleges experience significantly higher in quality and extent of skills training, receiving feedback, and active learning than the other types of early patient experience. The R/UOP immersion experience, although rated significantly lower than the Colleges in most clinical-skills areas, was rated significantly higher than limited community preceptorships in most categories across years. The Colleges engendered the greatest development of comfort in clinical settings in 2010 over all other settings, but there were no differences in 2011. The high ratings for all three settings suggest that all of the experiences advance comfort in clinical settings. In contrast, for understanding the life of a physician and what practice is like, both types of community preceptorships were rated significantly higher than the Colleges. R/UOP, in which students live in a community and are intensely involved for a month, was rated highest for understanding the life of a physician. Similarly, for career/specialty decisions, both R/UOP and preceptors showed clear advantages over the Colleges.

All three types of early patient experience appear to achieve their stated goals. Preceptorships do not appear to promote student comfort with patients to the same extent as R/UOP and the Colleges; this may be due to considerable shadowing during preceptorships rather than active engagement, as identified in the 2009 comparisons by students of preceptorships with Colleges. Students were, however, able to receive exposure to specialty and career roles through preceptorships, in which they joined practice types of their choice.

The R/UOP experience, in which students live and work in a community, appeared to have the strongest impact on understanding practice and the life of the physician. It also appeared to provide active learning with a focus on clinical skills and feedback. The concentrated day-to-day contact with a physician preceptor that characterizes R/UOP for a full month may provide greater familiarity and comfort between the student and preceptor, leading to more intensive focus on and involvement in teaching and learning.

These data provide guidance for medical schools as they look to desired and expected outcomes associated with early patient experiences. It is important to note that there was some variability in students’ responses to community preceptorships; some students described very active, hands-on community preceptorships. How-ever, the more common experience was shadowing with little active involvement. R/UOP also provided variable experiences, from shadowing to very active involvement, but there was more evidence of active learning, as indicated by significantly higher ratings in relevant categories than for preceptorships.

Only the Colleges had a structured, systematic approach to ensuring student responsibility at the bedside. Yardley and colleagues have made the important observation that learners actively influence learning environments just as learning environment actively influence learning; thus, experiential learning is located within bi-directional interactions [8]. In the Colleges model, students are active participants with defined roles that are preliminary to, yet moving toward, active roles as meaningful clinical providers. The R/UOP model has less structure than the Colleges but more immersion than preceptorships as a result of living in and being a guest member of the preceptor’s community. In both settings, students are able to influence their environment; in contrast, preceptorships appear to provide primarily passive shadowing without the ability of the student to influence his/her environment or develop meaningful relationships within that environment. However, students receive a “snapshot” of active physician life that can help with future decisions and understanding the roles of physicians.

As medical schools assess early patient contact, we recommend the following: Clinical-skills training may be maximized by structured experiences with faculty mentors dedicated to longitudinal teaching at the bedside in which students are assigned to specific roles with patients, including conduct of history, physical examination, preliminary differential diagnosis, oral case presentation, and write-ups. This appears to provide contact with patients, active learning, skills training, and acculturation to teams. For orienting students to physician life and specialty choices, preceptorships and R/UOP-type experiences provide insights into the life of a physician and potential career preferences. The R/UOP experience appears to be particularly valuable for understanding the life of a physician, as students see physicians in their community during and after hours, and develop a relationship with a preceptor that permits sustained learning, understanding and acculturation.

This study has several limitations. The study was conducted at a single large medical school and therefore the findings may not be generalizable to other medical schools. However, few medical schools provide multiple types of early patient experiences and this comparison, even if at a single site, provides valuable information. We were unable to assess whether students used early patient experiences to integrate knowledge and skills, as called for by the Carnegie report [1]. The most structured experience, the Colleges, is not formally tied to the classroom curriculum, although there is sometimes overlap. The other early patient contact experiences are unrelated to curriculum content. We also looked at a limited set of outcomes and did not assess potential affective outcomes. We wanted to assess areas common to the three settings that might be tied to concrete curricular goals.

## Conclusions

Different types and formats of early patient experiences may provide unique learning outcomes and acculturation for preclinical medical students. In developing medical school curricula, educators may benefit from assessing the objectives of offering patient experiences for preclinical students—whether clinical-skills development, acculturation to clinical settings, career exposure, or other—and plan patient experience formats accordingly. Careful attention might focus on the extent to which an active experience is desired for preclinical students; if active involvement is favored, more structured experiences like the Colleges and/or more intensive experiences like R/UOP may succeed better. Our data suggest that offering multiple types of early patient experiences may provide students with a broader set of concrete outcomes than a single type of early patient experience.

## Competing interests

The authors declare that they have no competing interests.

## Authors’ contributions

MDW conceptualized, designed and led all aspects of the study, including data collection, analyses, drafting and critically revising the manuscript. MBJ participated directly and actively in all of these aspects of the study. IW, PGR and AJJS were actively involved in analysis and interpretation of data and critical revision of the manuscript. All authors have approved this manuscript.

## Pre-publication history

The pre-publication history for this paper can be accessed here:

http://www.biomedcentral.com/1472-6920/13/80/prepub
